# See One, B1, Treat One: Identifying and Managing Thiamine Deficiency in a Patient With Altered Mental Status

**DOI:** 10.7759/cureus.100736

**Published:** 2026-01-04

**Authors:** Michael Freddy, Tyler B Larsen, Satya Patel

**Affiliations:** 1 Medicine, David Geffen School of Medicine, University of California Los Angeles, Los Angeles, USA; 2 Internal Medicine, David Geffen School of Medicine, University of California Los Angeles, Los Angeles, USA; 3 Internal Medicine, Veterans Affairs Greater Los Angeles Healthcare System, Los Angeles, USA; 4 Internal Medicine, University of California Los Angeles, Los Angeles, USA

**Keywords:** alcohol use disorder (aud), beriberi, gait ataxia, thiamine, wernicke-korsakoff syndrome

## Abstract

Nutritional deficiencies in the United States are relatively uncommon due to federal regulations surrounding the fortification of food. As a result, the clinical syndromes that result from nutritional deficiencies are not commonly seen, making them less likely to be encountered in the clinical setting. Thiamine deficiency in particular is an important nutritional deficiency to consider, as the presentation is highly variable and can include encephalopathy and multiple neurologic findings. We present a case of altered mental status that appears to be Wernicke’s encephalopathy due to the rapid improvement of symptoms with intravenous thiamine supplementation, corroborated by a low serum thiamine level. Assessing access to food is a critical piece of history in making this diagnosis, and early recognition can promote fully supplementing patients with thiamine to prevent ongoing thiamine deficiency and possible Korsakoff syndrome.

## Introduction

Thiamine deficiency is relatively uncommon in the United States due to fortification of foods with thiamine, but it remains an important diagnosis to consider, especially in patients on long-term loop diuretics, who have undergone bariatric surgery, and with alcohol use disorder [1-3]. Thiamine deficiency can present as wet beriberi or as one of three presentations of dry beriberi: Wernicke’s encephalopathy, Korsakoff syndrome, or a combination of peripheral neuropathy and glossitis [1,4]. Wernicke’s encephalopathy is an acute presentation of thiamine deficiency described with a classic triad of vision issues, gait imbalance, and altered mental status [1]. If left untreated, this can progress to a more permanent and severe form called Korsakoff syndrome [1].

Patients with thiamine deficiency rarely present with the classic presentation, and this report emphasizes the importance of having a high index of clinical suspicion for thiamine deficiency to diagnose it.

## Case presentation

A 76-year-old male with hypertension, hyperlipidemia, aortic stenosis status-post aortic valve replacement, and hypothyroidism was brought into the emergency room by the fire department after being found down.

In the emergency department, the patient was unable to recall the events leading to him being brought in, but he noted a two-year history of progressive bilateral lower extremity weakness and thigh pain that may have contributed to him losing his balance and falling to the ground without loss of consciousness or head trauma. Over the past week, he noted having some issues with memory problems, noting that for the first time, he had left his home and was unable to figure out how to return. He denied fevers, shortness of breath, or dysuria, but noted some urinary incontinence.

The patient was experiencing homelessness and was currently living in a hotel. He was unable to explain how he accesses meals, but noted drinking a few beers per week.

His vital signs were notable for a BP of 106/65 mmHg. Physical examination revealed a 2/6 systolic crescendo-decrescendo murmur loudest at the right second intercostal area, pain with palpation of the suprapubic area, bilateral lower extremity edema with deep purple hyperpigmentation, mild tenderness to the bilateral thighs, 5/5 strength throughout his bilateral upper and lower extremities, and a wide-based shuffling gate. The patient's cranial nerve exam was normal, and no nystagmus or ophthalmoplegia was noted. Extensive laboratory testing was performed upon admission (Table 1).

**Table 1:** Serum laboratory findings on admission

Serum Laboratory Study	Result	Reference Range
White blood cell	10.85 per uL	4.5 to 11.0 per uL
Blood urea nitrogen	53 mg/dL	5 to 25 mg/dL
Creatinine	1.56 mg/dL	0.52 to 1.28 mg/dL
Calculated estimated glomerular filtration rate	46 mL/min	88 to 110 mL/min
Calcium	8.8 mg/dL	8.4 to 10.2 mg/dL
Creatinine kinase	1584 U	40 to 280 U/L
Lactate	1.12 mmol/L	0.5 to 2.2 mmol/L
Thyroid-stimulating hormone	8.365 uIU/mL	0.55 to 4.78 uIU/mL
Free T4	1.21 ng/dL	0.66 to 1.73 ng/dL
Vitamin B12	3145 pg/mL	160 to 911 pg/mL
Folate	15.82 ng/mL	> 5.37 ng/mL
Rapid plasma reagin	Non-reactive	Non-reactive
Human immunodeficiency virus antibody screening	Negative	Negative

**Figure 1:**
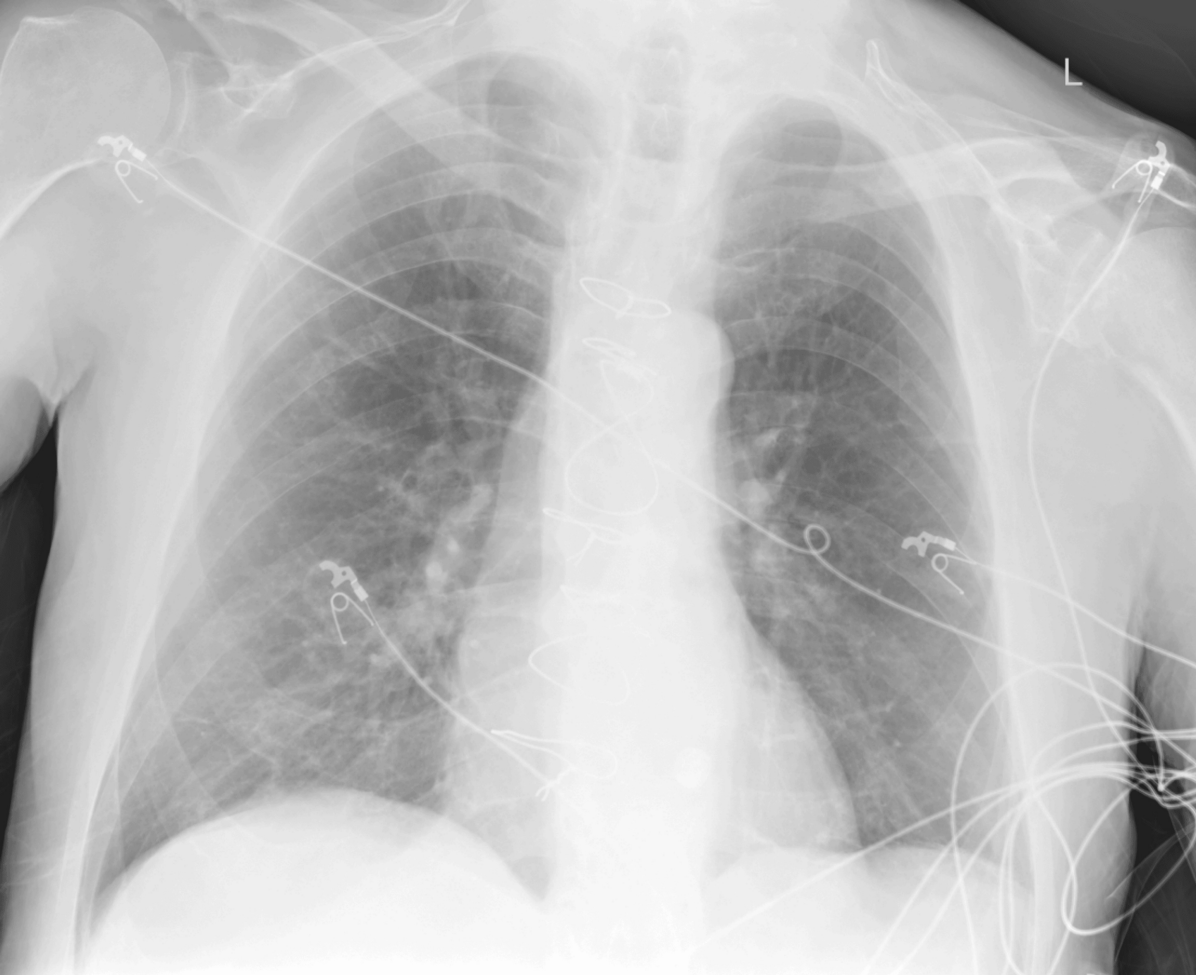
Portable chest X-ray showing no acute abnormalities

**Figure 2:**
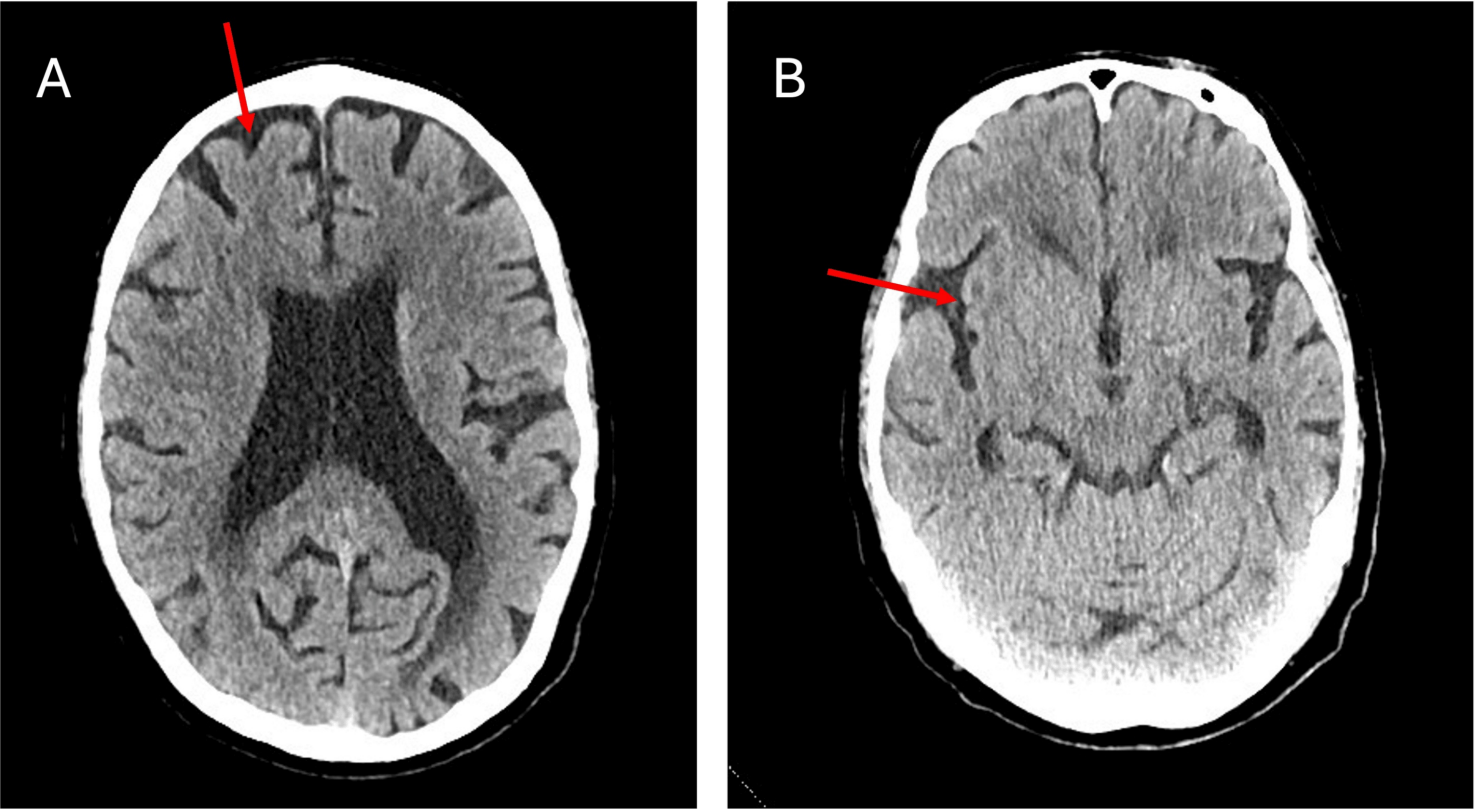
Computed tomography of the head without contrast on admission. A) The arrow indicates a deep sulcus. B) The arrow indicates an area of hyperintensity.

A portable chest X-ray revealed no acute cardiothoracic abnormality (Figure 1). A computed tomography (CT) scan of the head without contrast was performed, which showed mild-moderate global cerebral atrophy with a moderate degree of chronic microvascular ischemia (Figure 2).

A urinalysis and urine drug screen were ordered upon admission. A serum thiamine level was also ordered upon admission. The patient was given intravenous fluid and resuscitation for possible mild rhabdomyolysis and also empirically started on intravenous thiamine 500mg. Given the lack of urinary tract symptoms experienced by the patient, empiric antibiotics were not initiated (Table 2).

**Table 2:** Urine laboratory findings on admission

Urine Study	Result	Reference Range
Specific gravity	1.020	1.008 to 1.030
Protein	100 mg/dL	0 to 20 mg/dL
Ketones	20 mg/dL	Trace
Nitrites	Negative	Negative
Leukocyte esterase	500 per uL	0 to 25 per uL
White blood cell	>182 per high-powered field	0 to 5 per high-powered field
Red blood cell	123	0 to 5 per high-powered field
Squamous epithelial cells	Small	None to rare
Ethanol	Negative	Negative
Amphetamines	Negative	Negative
Benzodiazepines	Negative	Negative
Opiates	Negative	Negative

The following morning, repeat examination revealed that he was alert and oriented to self, place, time, and location, and was able to better recall his issues with ambulation. His repeat gait exam normalized. The urine culture resulted as *Staphylococcus aureus* 15,000 cfu/mL and *Aerococcus urinae* >100,000 cfu/mL. The thiamine level resulted as <6 mmol/L (normal 8 - 30 mmol/L). The results of the urine culture were thought to be asymptomatic bacteriuria, and as the patient did not develop additional urinary tract symptoms while off antibiotics, the decision to not give antibiotics was maintained. Given the severely reduced thiamine level, the decision was made to complete an additional two days of intravenous thiamine repletion. For the duration of his hospitalization, the patient’s mental status remained intact, and he did not report any urinary symptoms. Given that he was living independently, he was given extensive counseling and references to ensure that he maintained access to food. He was also given resources for further support in the event that he had difficulty completing activities of daily living and independent activities of daily living, especially in the context of his imaging findings.

## Discussion

As is the case with all vitamins, thiamine deficiency is caused by lack of access to thiamine, lack of absorption, and/or increased excretion. Lack of access to thiamine is relatively uncommon in the United States, as many foods are fortified with thiamine to prevent this [1]. Lack of absorption can occur in patients with alcohol use disorder, where active alcohol consumption downregulates expression of thiamine transporter-1 (THTR-1), which usually promotes thiamine reabsorption in the jejunal brush border [5]. Bariatric surgery can also alter gastric anatomy, thereby impairing enteral thiamine reabsorption [2]. As thiamine is a water-soluble vitamin, long-term loop diuretics can also cause thiamine deficiency [6].

Typically, a combination of the aforementioned pathologies can contribute to clinically significant thiamine deficiency in patients. To detect thiamine deficiency, clinicians must be aware of the classic and atypical presentations of thiamine deficiency, which is exceptionally challenging given how infrequently this diagnosis occurs in the United States due to the fortification of foods. Developing an illness script for thiamine deficiency and exploring lack of access to food during history taking can be ways to recognize this diagnosis.

Inherent to this illness script is understanding that in the typical work-up of altered mental status, the base rate of thiamine in the United States does not require it to be on the initial differential diagnosis and that more common causes of encephalopathy should instead be prioritized. In the case of this patient, especially in the context of being found down, a wide metabolic and infectious work-up was rightly prioritized, with metabolic causes outside of nutritional deficiencies being placed at the top of the differential diagnosis.

Hypothyroidism was on the initial differential diagnosis given the elevated thyroid-stimulating hormone (TSH); a TSH level less than 10 uIu/mL in conjunction with a normal free T4 level made subclinical hypothyroidism more likely. The patient’s initial urinary results were possibly suggestive of a urinary tract infection despite the absence of urinary tract symptoms. The patient’s brain imaging was consistent with early dementia in the right context. Rhabdomyolysis was also considered, given the elevation in creatine kinase; however, a much larger rise would be expected to lead to acute kidney injury from rhabdomyolysis and subsequent uremia to explain the altered mental status. Diagnostic clarity was obtained since the patient improved despite not receiving treatment for any of these diagnoses.

As with many patients, there are often multiple diagnoses contributing to a clinical syndrome, and this patient’s case is no different. Initial intracranial imaging shows findings that would be consistent with the mild cognitive impairment-dementia spectrum. Patients with dementia are not only more likely to suffer from nutritional deficiencies, but are also more likely to demonstrate clinical symptoms of nutritional deficiency, likely from dementia-related alterations in the metabolism and utilization of nutrients [7]. The additional layer of clinical complexity of this case was the initial decision to avoid giving antibiotics in the face of a possible urinary tract infection, instead electing to classify it as asymptomatic bacteriuria. There has been an emerging movement to avoid unnecessary antibiotics for bacteriuria in the absence of symptoms, which is challenging as older patients who present with altered mental status and are often unable to provide history or demonstrate exam findings consistent with symptomatic bacteriuria (namely dysuria and suprapubic tenderness). The decision not to give antibiotics in these cases must always remain individualized, as urinary tract infections can commonly cause encephalopathy and sepsis in older patients.

Thiamine deficiency can present as a cardiomyopathy with the clinical syndrome of wet beriberi [1]. Thiamine is a necessary cofactor to facilitate oxidative phosphorylation to produce energy for myocardial cells, and its absence can precipitate high-output heart failure [1]. As with most patients with heart failure, they are likely to receive loop diuretics. Loop diuretics will cause the loss of water-soluble vitamins, of which one is thiamine; this may exacerbate the thiamine deficiency that initially led to the heart failure. Wernicke’s syndrome is noted by the classic findings of encephalopathy, ataxic gait, and ophthalmoplegia; only 20% of patients present with the triad [8]. Korsakoff syndrome is thought to be a further progressed version of Wernicke’s, where patients develop anterograde amnesia, confabulation, and memory impairment. A subset of patients with thiamine deficiency will have neither wet beriberi nor Wernicke-Korsakoff syndrome and instead might have peripheral neuropathy and even painful glossitis [9].

Intravenous thiamine should be prioritized over oral repletion for patients with Wernicke’s encephalopathy, wet beriberi, severe alcohol use disorder, and patients who have undergone bariatric surgery (given impaired enteral absorption) [4].

This patient only had two of the findings of Wernicke’s syndrome, but rapid improvement after thiamine repletion suggests the diagnosis despite the patient having many other potential etiologies. As this is a rarer diagnosis in the United States, review of common etiologies of altered mental status, such as infection or common metabolic derangements, should be initially prioritized. If thiamine is to be empirically given, it is recommended to obtain a thiamine level prior to repletion, as the level will normalize as early as 6 hours after intravenous repletion [10].

## Conclusions

In summary, thiamine deficiency can present atypically and should be considered in cases of altered mental status, especially in patients with issues with enteral absorption (either through alcohol use disorder or through altered gastric anatomy). As with all cases of altered mental status, prioritizing the common causes of infectious and structural pathologies is appropriate. In suspected cases of thiamine deficiency, empiric thiamine repletion can lead to rapid improvement in symptoms.
